# Variation of diagnosis and treatment of catheter-associated urinary tract infections: an online survey among caretakers involved

**DOI:** 10.1177/17562872231191305

**Published:** 2023-09-06

**Authors:** Tess van Doorn, Sophie A. Berendsen, Rosa L. Coolen, Jeroen R. Scheepe, Bertil F. M. Blok

**Affiliations:** Department of Urology, Erasmus MC, Dr Molewaterplein 40, Rotterdam 3015 GD, The Netherlands; Department of Urology, Erasmus Medical Center, Rotterdam, The Netherlands; Department of Urology, Erasmus Medical Center, Rotterdam, The Netherlands; Department of Urology, Erasmus Medical Center, Rotterdam, The Netherlands; Department of Urology, Erasmus Medical Center, Rotterdam, The Netherlands

**Keywords:** bacteriuria, caretakers, catheter, clean intermittent catheterization, general practitioner, indwelling catheter, rehabilitation medicine, self-catheterization, urinary tract infection, urology

## Abstract

**Background:**

The diagnosis of a clinically significant catheter-associated urinary tract infection (CAUTI) in patients performing clean intermittent catheterization (CIC) or with an indwelling catheter (IC) can be challenging.

**Objective:**

To get an insight into the variation of the used definition, diagnosis and management of CAUTIs by relevant healthcare workers in the Netherlands.

**Design:**

An online clinical scenario-based survey.

**Methods:**

The survey was built in Limesurvey and distributed to healthcare workers from randomly selected urology departments, rehabilitation departments/centres and general practice offices between January and May 2022. Questions regarding their field of experience, management strategies, used guidelines and two hypothetical cases with clinical scenarios of a possible CAUTI were included.

**Results:**

A total of 172 individuals participated, of which 112 completed the survey. In all, 32 individuals who completed the survey partially were also included. Participants consisted of 68 [44 urologists, 22 rehabilitation doctors (RDs) and 2 general practitioners (GPs)] doctors, 60 nurses (46 from the urology department and 14 from rehabilitation centres/departments) and 16 medical assistants (13 from urology department and 3 from GP offices). The majority consulted patients with an IC or on CIC on a daily/weekly or monthly basis. In all, 35 urologists (79.5%), 9 RDs (40.9%), 21 (45.7%) nurses in the urology department and 6 (42.9%) nurses from a rehabilitation department/centre indicated bladder irrigation as a treatment option for prevention/treatment of CAUTIs, treatment of symptoms or treatment of blockage of the catheter. In the clinical scenarios presented, treatment discrepancies were seen between subspecialties and healthcare workers. Various guidelines were named for the definition of CAUTIs.

**Conclusion:**

A considerable variation in diagnoses and management of CAUTIs between the healthcare workers involved was seen. Uniformity in diagnosing and managing CAUTIs, to prevent overtreatment and possible resistance to antibiotics, is advised. Suitable multidisciplinary guidelines are preferred.

## Introduction

In the Netherlands, there were approximately 45,000 extramural patients on clean intermittent catheterization (CIC) and 54,000 extramural patients with an indwelling catheter (IC) in 2018.^1,2^

One month after initiating CIC or insertion of an IC, almost 100% of these patients will have bacteriuria.^3^ Many patients test their urine at their general practitioner (GP) or an outpatient clinic when they suspect a urinary tract infection (UTI), often based on an altered aspect or foul smell of urine, increased leakage of urine, urge to void or lower abdominal pain. In common practice, urine will be analysed, and bacteria and leukocytes will be present due to the (intermittent) catheter. Antibiotics will be prescribed, despite the lack of distinctive alarm symptoms for UTI, like fever or cognitive changes. The annual incidence of such antibiotic prescription has been reported to be 20% for CIC and 57% for an IC.^4,5^ Resistance to antibiotics is a worldwide problem, especially in patient populations that receive antibiotics regularly. Due to the overtreatment of patients on CIC or with an IC, an increased antimicrobial resistance is seen a less treatment options are available.^6–8^

The definition and diagnosis of a catheter-associated UTI (CAUTI) in patients performing CIC or with an IC can be challenging. A previous study on the diagnosis of CAUTI in paediatric patients with a neurogenic bladder requiring CIC showed a variation in physicians’ opinions regarding the diagnosis.^9^ Recently, an editorial on uropathogens, their pathogenesis mechanisms, immunogenetics, virulence and predisposing factors in the context of ‘host–pathogen interactions’ was published. These more basic and objective measurements and information on pathogens can deepen our understanding of the nature of infectious agents and possible resistance to antibiotics. In future perspective, this can lead to more research possibilities for innovative therapeutic strategies and reducing inaccurate treatment of (CA)UTIs when incorporated in a multidisciplinary guideline.^10^

The primary objective is to obtain an insight into the variation of clinical practice used for CAUTIs between subspecialists, nurses and supporting employees (medical assistants) involved in the Dutch healthcare system. Secondary objectives are the evaluation of the work experience of participants and how familiar they are with patients on CIC or an IC. This will give an understanding of the impact and challenges of diagnosing CAUTIs in clinical practice.

Our hypothesis is that a considerable variation in interpretation of the definition and diagnosis will be found among healthcare workers involved in the field of urology, rehabilitation medicine and general practices.

## Material and methods

### Survey development

**Figure 1.**
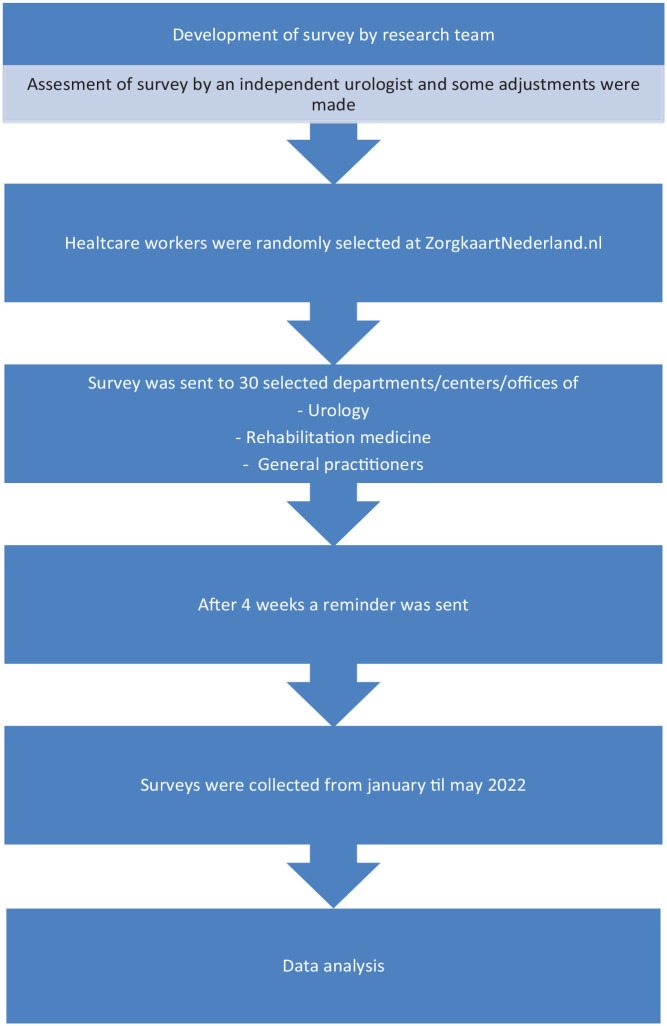
Flow chart of the study procedure.

This survey was developed by the research team, consisting of three clinically trained researchers and two urologists, and was based on their clinical experience. A few adjustments were made after an independent urologist was consulted on the distinctiveness of the questions. The questionnaire consists of multiple choice questions regarding the field of work, work experience and two hypothetical cases with two/three clinical scenarios of a possible CAUTI. Three comparable Dutch questionnaires were created, one for doctors, one for rehabilitation/urology nurses and one for medical assistants. The translated questionnaires are attached as Supplemental Materials. An overview of the clinical scenarios and answer options is provided in Table 1. A schematic overview of the study procedure is shown in Figure 1.

**Table 1.** Clinical cases/scenarios and different answer options per healthcare worker involved.

Clinical cases/scenarios	Answers doctors	Answers (continence) nurses	Answers medical assistants
Case 1: An 85-year-old man cannot undergo surgery for his BPH due to his cardiac condition and is therefore dependent on intermittent self-catheterization. *Situation 1*: The urine has been cloudy for a week and the left flank is tender. He also suffers from general malaise. That morning, the temperature is 37.4°C. Urine analysis shows 3+ leukocytes and 2+ erythrocytes. *Situation 2*: The urine has been cloudy for 3 days and the man has complaints of increasing incontinence between catheterizations and an increased sense of urgency, which necessitates a catheterization frequency of 10 times/day. That morning, the temperature is 37.4°C. Urine analysis shows 3+ leukocytes and 2+ erythrocytes.	*What would be your next step?* a. Wait b. Take a UC and wait c. Take a UC and start with antibiotics d. Take a UC and start antibiotics after the results e. The result of the urine analysis is sufficient, start with antibiotics f. Otherwise, namely. . .	*What would be your next step?* a. Wait b. Take a UC and wait c. Take a UC and start with antibiotics d. Take a UC and start antibiotics depending on the results e. Schedule a consultation with a nurse at the outpatient clinic f. Otherwise, namely. . .	*What would be your next step?* a. Reassure the patient and wait b. Schedule an appointment with the doctor c. Refer patient to GP/specialist d. Schedule an appointment with the doctor and take a urinalysis e. Consult the doctor for further policy f. Otherwise, namely. . .
Case 2: A 42-year-old woman has a SPC for a spinal cord injury. She is known to have bacteria in her urine. *Situation 1*: The woman has been suffering from increasing spasms in the legs for 3 days and a temperature of 38.2°C has been measured twice. The urine is dark and cloudy, despite the fact that the woman has been drinking well over the past few days. Urine analysis shows 2+ leukocytes and 1+ erythrocytes. *Situation 2*: The woman has been suffering from flaky and strong-smelling urine for 1.5 weeks. The patient does not feel ill otherwise. Because a holiday to France is planned for next week, she is asking for antibiotics. Urine analysis shows 2+ leukocytes and 1+ erythrocytes. *Situation 3*: The woman has been experiencing leakage along the catheter for a week. The patient does not feel ill, she only suffers from headache during the leakage. Urine analysis shows 2+ leukocytes and 1+ erythrocytes.			

BPH, benign prostatic hyperplasia; GP, general practitioner; SPC, suprapubic catheter.

### Selection of healthcare workers

Participants in this survey were the following healthcare workers involved in the care of patients on CIC or with an IC: GPs, rehabilitation doctors (RDs), urologists, (continence) nurses and medical assistants. We aimed to include 20 participants per subcategory. Healthcare workers were randomly selected at zorgkaartnederland.nl (ZorgkaartNederland). This is an independent website of the Dutch Patient Federation, where patients can share their experiences and valuation of individual healthcare workers or healthcare facilities.

### Survey acquisition

The survey was built in LimeSurvey (LimeSurvey GmBH, Hamburg, Germany), an online, open-sourced survey application. Between January and May 2022, an invitation for participation was sent by email to the selected urology departments, rehabilitation departments/centres and GP offices. The invitation contained an explanation and a direct hyperlink to the survey. Four weeks after the first invitation, a reminder was sent. All data were collected anonymously, the invitation stated informed consent was given by filling in the survey. Questionnaires were excluded if ⩾50% of the questions were left open.

### Study parameters

The main study parameter is to indicate variations of diagnosis and treatment policies of CAUTI in patients on CIC or with an IC between healthcare workers involved. The hypothesis is that there is no conclusive definition and management strategy used for this population regarding bacteriuria and CAUTIs, leading to variations in clinical practice. An overview of all outcome measures collected is shown in Supplemental Material 2.

### Statistics

Descriptive statistics will be used to describe the baseline characteristics of participating healthcare workers. Binomial or categorical outcome measures will be analysed using chi-square tests and quantitative outcome variables by *t*-tests or Mann–Whitney U tests.

## Results

A total of 172 individuals participated, of which 112 completed the survey. In all, 28 participants were excluded, due to not completing >50% of the questionnaire. The remaining 32 participants who completed the survey partially were also included in the analysis. The 144 participants consisted of 68 doctors, 60 nurses and 16 medical assistants. Further characteristics are provided in Table 2. Due to a low number of responses from GP offices, these answers are not included in the table but are written out in the results section.

**Table 2.** Characteristics of participants.

Urology	Physician (*n* = 44)	Doctors’ assistant/secretary (*n* = 13)	(Continence) nurse (*n* = 46)	Rehabilitation medicine	Physician (*n* = 22)	(Continence) nurse (*n* = 14)
Working environment						
Academic hospital	14 (31.8%)	0	1 (2.2%)		1 (4.5%)	0
Non-academic hospital	29 (65.9%)	13 (100%)	44 (95.6%)		5 (22.7%)	0
Rehabilitation centre	–	–	–		13 (59.1%)	14 (100%)
Other	0	0	1 (2.2%)		2 (9.1%)	0
Working experience (years)						
<5	9 (20.5%)	5 (38.5%)	7 (15.2%)		7 (31.8%)	6 (42.9%)
5–10	22 (50%)	4 (30.8%)	3 (6.5%)		10 (45.5%)	2 (14.3%)
10–15	9 (20.5%)	0	8 (17.4%)		0	2 (14.3%)
>15	4 (9.1%)	3 (23.1%)	28 (60.9%)		5 (22.7%)	4 (28.6%)
Incidence CIC						
Daily	18 (40.9%)	6 (46.2%)	18 (39.1%)		7 (31.8%)	2 (14.3%)
Weekly	23 (52.9%)	7 (53.9%)	28 (60.9%)		4 (18.2%)	3 (21.4%)
Monthly	3 (6.8%)	0	0		6 (27.3%)	2 (14.3%)
Annually	0	0	0		5 (22.7%)	7 (50%)
Never	0	0	0		0	0
Incidence IC						
Daily	20 (45.5%)	9 (69.2%)	34 (73.9%)		6 (27.3%)	3 (21.4%)
Weekly	21 (47.7%)	4 (30.8%)	12 (26.1%)		5 (22.7%)	2 (14.3%)
Monthly	1 (2.3%)	0	0		10 (45.5%)	5 (35.7%)
Annually	1 (2.3%)	0	0		1 (4.5%)	4 (28.6%)
Never	0	0	0		0	0

Due to the low number of responses from the GP offices, these answers are not included in the table but can be found in the results section of this article.

### Characteristics and working experience

#### Physicians

In all, 44 of the participating physicians were urologists and 22 RDs. In total, 14 urologists worked in an academic hospital and 29 in a non-academic hospital. The majority (*n* = 13, 59.1%) of RDs worked in rehabilitation centres, one (4.5%) was working in an academic hospital and five (22.7%) in non-academic hospitals. Two RDs stated to work elsewhere, one in a geriatric rehabilitation centre and one in a nursing home. Most urologists stated to see patients who perform CIC on a daily (*n* = 18, 40.9%%) or weekly (*n* = 23, 52.3%) basis and patients with an IC daily (*n* = 20, 45.5%) or weekly (*n* = 21, 47.7%). Most RDs stated to see patients on CIC every day (*n* = 7, 31.8%) and patients with an IC monthly (*n* = 10, 45.5%). One GP had a working experience of 5–10 years and one of >15 years. One GP stated to never see patients on CIC or with an IC one GP stated to see both on a monthly basis.

#### Nurses

The majority of nurses were working in urology departments (*n* = 46, 76.7%), the other 23.3% (*n* = 14) were working in rehabilitation centres/departments. Of nurses working in a urology department, 95.6% were in a non-academic hospital. Most continence nurses from urology departments had >15 years of working experience (60.9%, *n* = 28). In rehabilitation centres, most nurses (*n* = 6, 42.9%) had working experience of less than 5 years. Sixty percent of continence nurses from urology departments stated to see patients on CIC weekly (*n* = 28) and 73.9% of patients with an IC daily (*n* = 34). Nurses working in rehabilitation centres stated to see patients on CIC annually (*n* = 7, 50%) and patients with an IC monthly (*n* = 5, 35.7%) or annually (*n* = 4, 28.6%).

#### Medical assistants

A total of 16 medical assistants participated in this survey, 13 of them were working in urology departments and three in a GP office. All medical assistants in urology departments were working in a non-academic hospital, and most of them (*n* = 5, 38.5%) had working experience of <5 or 5–10 years (*n* = 4, 30.8%). They were in contact with patients on CIC daily (*n* = 6, 46.2%) or weekly (*n* = 7, 53.9%) and patients with an IC also daily (*n* = 9, 69.2%) or weekly (*n* = 4, 30.8%).

In GP offices, one medical assistant had a working experience of 5–10 years, one of 10–15 years and one of >15 years. One medical assistant stated to never see patients on CIC or with an IC, one stated to see both on a monthly basis and one patient with an IC monthly and patients on CIC yearly.

### Management of CAUTIs

#### Physicians

Most urologists (*n* = 35, 79.5%) indicate bladder irrigation (BI) as a treatment option when a patient experiences symptoms of a (possible) CAUTI. The main reasons to do so were treatment of catheter blockage (*n* = 28, 63.6%) or prevention of CAUTIs (*n* = 20, 45.5%). Two urologists (4.5%) named other reasons: prevention of stone formation and washing out debris. Gentamycin (*n* = 12, 27.3%) and NaCl (*n* = 10, 22.7%) were the most used irrigation substances by urologists. One urologist named another substance: iAluRil. Results are shown in Table 3.

**Table 3.** Incidence of BI as a treatment option, reasons why and used substances.

Urology	Physician (*n* = 44)	(Continence) nurse (*n* = 46)	Rehabilitation medicine	Physician (*n* = 22)	(Continence) nurse (*n* = 14)
Starting BI					
Yes	35 (79.5%)	21 (45.7%)		9 (40.9%)	6 (42.9%)
No	9 (20.5%)	24 (52.2%)		13 (59.1%)	8 (57.1%)
Reason BI					
Prevention CAUTIs	20 (45.5%)	5 (10.9%)		4 (18.2%)	2 (14.3%)
Treatment CAUTIs	6 (13.6%)	7 (15.2%)		0	0
Treatment symptoms	14 (31.8%)	16 (34.8%)		4 (18.2%)	3 (21.4%)
Treatment blockage	28 (63.6%)	12 (26.1%)		7 (31.8%)	4 (28.6%)
Other, namely. . .	2 (3) (4.5%)	5 (10.9%)		1 (4.5%)	2 (14.3%)
Type of BI					
Tap water	4 (9.1%)	0		2 (9.1%)	0
NaCl	10 (22.7%)	1 (2.2%)		3 (13.6%)	2 (14.3%)
Solutio G/Solutio R	6 (13.6%)	0		2 (9.1%)	1 (7.1%)
GAG-layer repairing irrigation	5 (11.4%)	2 (4.3%)		0	0
Gentamycin	12 (27.3%)	2 (4.3%)		0	0
Povidone iodine	0	0		0	0
Other, namely. . .	1 (2) (2.3%)	2 (4.3%)		0	0

CAUTIs, catheter-associated urinary tract infections; BI, bladder irrigation.

Nine RDs (40.9%) stated to use BI as a treatment option for a (possible) CAUTI in their patient population. Treatment of catheter blockage (*n* = 7, 31.8%) was the main reason to do so. One RD named another reason, namely for administration of medicine like anticholinergics or gentamycin. NaCl was the most used (*n* = 3, 13.6%) substance for irrigation by RDs.

One of the two GPs stated to use BI as a treatment option and one stated to never start BI. The reasons named was for treatment of the CAUTI itself and for prevention of catheter blockage. The question on which substance the GP used was left open.

#### Nurses

Of continence nurses working in urology departments, the distribution of nurses stating to start BI and nurses who stated not to do so was almost 50/50. The main reason for starting BI was the treatment of symptoms (*n* = 16, 34.8%). Other reasons given were because the physician said to do so (*n* = 3, 6.5%), when antibiotic treatment is no option (*n* = 1, 2.2%) and after an ileocystoplasty (*n* = 1, 2.2%). Only seven continence nurses gave answers to the question what type of BI they used, and these answers are shown in Table 3. Two continence nurses stated to use a different type of irrigation, both named polihexanid.

Six (42.9%) nurses working in rehabilitation departments stated to start with BI, mainly to treat blockage of the catheter (*n* = 4, 28.6%) or treatment of symptoms (*n* = 3, 21.4%). One nurse stated to do so when doctors advised to start, and one stated to start BI to prevent encrustation of the catheter, bladder stone formation and to wash out debris. Two (14.3%) continence nurses use NaCl for the irrigation and one (7.1%) Solutio G/Solutio R.

### Clinical scenarios

#### Physicians

The clinical scenarios and answer options are provided in Table 4(a–c). In the first situation of case 1, most urologists (*n* = 20, 48.8%) and RDs (*n* = 14, 73.7%) choose the answer option to take a urine culture (UC) and to start with antibiotics. One of the GPs stated to take a culture and wait, one to take a culture and start antibiotics after the results were available. Two urologists gave different answer options, advising to increase the CIC frequency and fluid intake.

**Table 4.** (a) Clinical scenarios of physicians.: U stands for urologists (*n* = 41), RD stands for rehabilitation doctors (*n* = 19) and GP stand for general practitioners (*n* = 2).

Clinical scenario	Wait	Take a UC and wait	Take a UC and start with antibiotics	Take a UC and start antibiotics after the results	The result of the urine analysis is sufficient, start with antibiotics	Take a UC and start with BI	Other, namely. . .	
Case 1: An 85-year-old man cannot undergo surgery for his BPH due to his cardiac condition and is therefore dependent on CIC. *Situation 1*: The urine has been cloudy for a week and the left flank is tender. He also suffers from general malaise. That morning, the temperature is 37.4°C. Urine analysis shows 3+ leukocytes and 2+ erythrocytes.	U: *n* = 1, 2.4% RD: *n* = 0 GP: *n* = 0	U: *n* = 8, 19.5% RD: *n* = 2, 10.5% GP: *n* = 1, 50%	U: *n* = 20, 48.8% RD: *n* = 14, 73.7% GP: *n* = 0	U: *n* = 10, 24.4% RD: *n* = 2, 10.5% GP: *n* = 1, 50%	U: *n* = 0 RD: *n* = 0 GP: *n* = 0	U: *n* = 0 RD: *n* = 0 GP: *n* = 0	U: *n* = 2, 4.9% RD: *n* = 1, 5.3% GP: *n* = 0	
*Situation 2*: The urine has been cloudy for 3 days and the man has complaints of increasing incontinence between catheterizations and an increased sense of urgency, which necessitates a catheterization frequency of 10 times/day. That morning, the temperature is 37.4°C. Urine analysis shows 3+ leukocytes and 2+ erythrocytes.	U: *n* = 0 RD: *n* = 0 GP: *n* = 0	U: *n* = 5, 12.2% RD: *n* = 3, 17.6% GP: *n* = 2	U: *n* = 22, 53.7% RD: *n* = 10, 58.8% GP: *n* = 0	U: *n* = 10, 24.4% RD: *n* = 0 GP: *n* = 0	U: *n* = 1, 2.4% RD: *n* = 2, 11.8% GP: *n* = 0	U: *n* = 0 RD: *n* = 0 GP: *n* = 0	U: *n* = 3, 7.3% RD: *n* = 2, 11.8% GP: *n* = 0	
								Would you change the catheter?
Case 2: A 42-year-old woman has a SPC for SCI. She is known to have bacteria in her urine. *Situation 1*: The woman has been suffering from increasing spasms in the legs for 3 days and a temperature of 38.2°C has been measured twice. The urine is dark and cloudy, despite the fact that the woman has been drinking well over the past few days. Urine analysis shows 2+ leukocytes and 1+ erythrocytes.	U: *n* = 0 RD: *n* = 0 GP: *n* = 0	U: *n* = 5, 13.9% RD: *n* = 0 GP: *n* = 0	U: *n* = 20, 55.6% RD: *n* = 9, 64.3% GP: *n* = 2, 100%	U: *n* = 4 RD: *n* = 2, 14.2% GP: *n* = 0	U: *n* = 0 RD: *n* = 0 GP: *n* = 0	U: *n* = 3, 8.3% RD: *n* = 2, 14.2% GP: *n* = 0	U: *n* = 4, 11.1% RD: *n* = 1, 7.1% GP: *n* = 0	U yes: 14 (38.9%) U no: 22 (61.1%) RD yes: 13 (92.9%) RD no: 1 (7.1%) GP yes: 1 (50%) GP no: 1 (50%)
*Situation 2*: The woman has been suffering from flaky and strong-smelling urine for 1.5 weeks. The patient does not feel ill otherwise. Because a holiday to France is planned for next week, she is asking for antibiotics. Urine analysis shows 2+ leukocytes and 1+ erythrocytes.	U: *n* = 8, 23.5% RD: *n* = 1, 7.1% GP: *n* = 0	U: *n* = 9, 26.5% RD: *n* = 7, 50% GP: *n* = 0	U: *n* = 1, 2.9% RD: *n* = 0 GP: *n* = 1, 50%	U: *n* = 2, 5.9% RD: *n* = 0 GP: *n* = 0	U: *n* = 0 RD: *n* = 0 GP: *n* = 0	U: *n* = 9, 26.5% RD: *n* = 5, 35.7% GP: *n* = 0	U: *n* = 5, 14.7% RD: *n* = 1, 7.1% GP: *n* = 1, 50%	U yes: 1 (2.9%) U no: 33 (97.1%) RD yes: 2 (14.3%) RD no: 12 (85.7%) GP yes: 1 (50%) GP no: 1 (50%)
*Situation 3*: The woman has been experiencing leakage along the catheter for a week. The patient does not feel ill, she only suffers from headache during the leakage. Urine analysis shows 2+ leukocytes and 1+ erythrocytes.	U: *n* = 4, 11.4% RD: *n* = 0 GP: *n* = 1, 50%	U: *n* = 9, 25.7% RD: *n* = 6, 42.9% GP: *n* = 0	U: *n* = 0 RD: *n* = 3, 21.4% GP: *n* = 0	U: *n* = 5, 14.3% RD: *n* = 0 GP: *n* = 0	U: *n* = 0 RD: *n* = 0 GP: *n* = 0	U: *n* = 11, 31.4% RD: *n* = 3, 21.4% GP: *n* = 1, 50%	U: *n* = 6, 17.1% RD: *n* = 2, 14.3% GP: *n* = 0	U yes: 9 (25.7%) U no: 26 (74.3%) RD yes: 7 (50%) RD no: 7 (50%) GP yes: 2 (100%) GP no: 0
(b) Clinical scenarios (continence) nurses: U stands for (continence) nurses from the urology department (*n* = 39) and R stands for (continence) nurses from the rehabilitation centre/department (*n* = 12).								
Clinical scenario	Wait	Take a UC and wait	Take a UC and direct consultation with the physician	Refer the patient to the GP/treating physician	Schedule a consultation with a nurse at the outpatient clinic	Other, namely. . .		
Case 1: An 85-year-old man cannot undergo surgery for his BPH due to his cardiac condition and is therefore dependent on CIC. *Situation 1*: The urine has been cloudy for a week and the left flank is tender. He also suffers from general malaise. That morning, the temperature is 37.4°C. Urine analysis shows 3+ leukocytes and 2+ erythrocytes.	U: *n* = 0 R: *n* = 0	U: *n* = 5, 12.2% R: *n* = 0	U: *n* = 31, 75.6% R: *n* = 8, 66.7%	U: *n* = 2, 4.9% R: *n* = 4, 33.3%	U: *n* = 1, 2.4% R: *n* = 0	U: *n* = 2, 4.9% R: *n* = 0		
*Situation 2*: The urine has been cloudy for 3 days and the man has complaints of increasing incontinence between catheterizations and an increased sense of urgency, which necessitates a catheterization frequency of 10 times/day. That morning, the temperature is 37.4°C. Urine analysis shows 3+ leukocytes and 2+ erythrocytes.	U: *n* = 0 R: *n* = 0	U: *n* = 5, 12.5% R: *n* = 1, 8.3%	U: *n* = 23, 57.5% R: *n* = 7, 58.3%	U: *n* = 2, 5% R: *n* = 2, 16.7%	U: *n* = 3, 7.5% R: *n* = 1, 8.3%	U: *n* = 7, 17.5% R: *n* = 1, 8.3%		
							Would you change the catheter?	
Case 2: A 42-year-old woman has a SPC for SCI. She is known to have bacteria in her urine. *Situation 1*: The woman has been suffering from increasing spasms in the legs for 3 days and a temperature of 38.2°C has been measured twice. The urine is dark and cloudy, despite the fact that the woman has been drinking well over the past few days. Urine analysis shows 2+ leukocytes and 1+ erythrocytes.	U: *n* = 0 R: *n* = 0	U: *n* = 1, 2.6% R: *n* = 0	U: *n* = 29, 76.3% R: *n* = 8, 80%	U: *n* = 1, 2.6% R: *n* = 1, 10%	U: *n* = 4, 10.5% R: *n* = 0	U: *n* = 3, 7.9% R: *n* = 1, 10%	U yes: 24 (63.2%) U no: 14 (36.8%) R yes: 7 (70%) R no: 3 (30%)	
*Situation 2*: The woman has been suffering from flaky and strong-smelling urine for 1.5 weeks. The patient does not feel ill otherwise. Because a holiday to France is planned for next week, she is asking for antibiotics. Urine analysis shows 2+ leukocytes and 1+ erythrocytes.	U: *n* = 4, 10.8% R: *n* = 1, 10%	U: *n* = 10, 27% R: *n* = 0	U: *n* = 10, 27% R: *n* = 5, 50%	U: *n* = 3, 8.1% R: *n* = 3, 30%	U: *n* = 3, 8.1% R: *n* = 1, 10%	U: *n* = 7, 18.9% R: *n* = 0	U yes: 10 (25.6%) U no: 29 (74.4%) R yes: 2 (20%) R no: 8 (80%)	
*Situation 3*: The woman has been experiencing leakage along the catheter for a week. The patient does not feel ill, she only suffers from headache during the leakage. Urine analysis shows 2+ leukocytes and 1+ erythrocytes.	U: *n* = 4, 10.5% R: *n* = 2, 20%	U: *n* = 9, 23.7% R: *n* = 1, 10%	U: *n* = 6, 15.8% R: *n* = 2, 20%	U: *n* = 4, 10.5% R: *n* = 2, 20%	U: *n* = 9, 23.7% R: *n* = 0	U: *n* = 6, 15.8% R: *n* = 3, 30%	U yes: 17 (45.9%) U no: 20 (54.1%) R yes: 3 (30%) R no: 7 (70%)	
(c) Clinical scenarios medical secretaries/assistants: U: stands for medical secretaries/assistants working in the urology department (*n* = 13), GP: stands for medical secretaries/assistants working in the GP office (*n* = 3).								
Clinical scenario	Reassure the patient and wait	Schedule an appointment with the physician	Refer the patient to the GP/treating physician	Schedule an appointment with the physician and do a urine analysis	Consulting the physician for further actions	Other, namely. . .		
Case 1: An 85-year-old man cannot undergo surgery for his BPH due to his cardiac condition and is therefore dependent on CIC. *Situation 1*: The urine has been cloudy for a week and the left flank is tender. He also suffers from general malaise. That morning, the temperature is 37.4°C. Urine analysis shows 3+ leukocytes and 2+ erythrocytes.	U: *n* = 0 GP: *n* = 0	U: *n* = 0 GP: *n* = 0	U: *n* = 0 GP: *n* = 0	U: *n* = 2, 18.2% GP: *n* = 0	U: *n* = 5, 45.5% GP: *n* = 2, 66.6%	U: *n* = 4, 36.4% GP: *n* = 1, 33.3%		
*Situation 2*: The urine has been cloudy for 3 days and the man has complaints of increasing incontinence between catheterizations and an increased sense of urgency, which necessitates a catheterization frequency of 10 times/day. That morning, the temperature is 37.4°C. Urine analysis shows 3+ leukocytes and 2+ erythrocytes.	U: *n* = 0 GP: *n* = 0	U: *n* = 0 GP: *n* = 0	U: *n* = 0 GP: *n* = 0	U: *n* = 2, 18.2% GP: *n* = 0	U: *n* = 5, 45.5% GP: *n* = 2, 66.6%	U: *n* = 4, 36.4% GP: *n* = 1, 33.3%		
Case 2: A 42-year-old woman has a SPC due to a spinal cord injury. She is known to have bacteria in her urine. *Situation 1*: The woman has been suffering from increasing spasms in the legs for 3 days and a temperature of 38.2°C has been measured twice. The urine is dark and cloudy, despite the fact that the woman has been drinking well over the past few days. Urine analysis shows 2+ leukocytes and 1+ erythrocytes.	U: *n* = 0 GP: *n* = 0	U: *n* = 0 GP: *n* = 0	U: *n* = 0 GP: *n* = 0	U: *n* = 3, 30% GP: *n* = 1, 33.3%	U: *n* = 5, 50% GP: *n* = 1, 33.3%	U: *n* = 2, 20% GP: *n* = 1, 33.3%		
*Situation 2*: The woman has been suffering from flaky and strong-smelling urine for 1.5 weeks. The patient does not feel ill otherwise. Because a holiday to France is planned for next week, she is asking for antibiotics. Urine analysis shows 2+ leukocytes and 1+ erythrocytes.	U: *n* = 0 GP: *n* = 0	U: *n* = 0 GP: *n* = 0	U: *n* = 0 GP: *n* = 0	U: *n* = 2, 20% GP: *n* = 0	U: *n* = 5, 50% GP: *n* = 2, 66.6%	U: *n* = 3, 30% GP: *n* = 1, 33.3%		
*Situation 3*: The woman has been experiencing leakage along the catheter for a week. The patient does not feel ill, she only suffers from headache during the leakage. Urine analysis shows 2+ leukocytes and 1+ erythrocytes.	U: *n* = 0 GP: *n* = 0	U: *n* = 0 GP: *n* = 0	U: *n* = 0 GP: *n* = 0	U: *n* = 0 GP: *n* = 0	U: *n* = 7, 70% GP: *n* = 2, 66.6%	U: *n* = 3, 30% GP: *n* = 1, 33.3%		

CIC, clean intermittent catheterization; BPH, benign prostatic hyperplasia; GP, general practitioner; SPC, suprapubic catheter; UC, urine culture.

In the second situation for case one, again most urologists (*n* = 22, 53.7%) and RDs (*n* = 10, 58.8%) stated to take a UC and to start with antibiotics directly. Both GPs stated to take a culture and wait. In the open answer section, two urologists stated to do a culture and start with antibiotics based on the results of previous cultures and one stated to rule out if a stone is present. One RD would give the patient an IC.

For the first situation of case two, most urologists (*n* = 20, 55.6%), RDs (*n* = 9, 64.3%) and both GPs (100%) would take a UC and start with antibiotics straight away. Two urologists answered to do a UC and start antibiotics after ruling out different explanations for the fever/spasms and one urologist stated to do a UC, start with BI and start with antibiotics based on the results of previous cultures. The last urologist stated not performing urine analysis when a patient has an IC or is on CIC, but taking a UC and ruling out if a stone is present. Most urologists (61.1%) stated not changing the catheter, and almost all RDs (92.9%) stated to do so.

In the second scenario of case 2, urologists were more divided as follows: eight (23.5%) stated to wait, nine (26.5%) stated to take a UC and wait and nine (26.5%) would take a UC and start with BI. Other given answers were as follows: three urologists would wait and advise the patients to increase their fluid intake and two would take a UC, start with BI and would give a prescription for antibiotics if necessary to take on holiday. Seven RDs (50%) stated to take a UC and wait and five (35.7%) would take a UC and start with BI. One GP stated to only start with BI with no further actions. In this scenario, most urologists (97.1%) and RDs (85.7%) stated not changing the catheter.

In the last scenario of case 2, most urologists (*n* = 11, 31.4%) stated to take a culture and start BI. Other answers given by the urologist included the following: only starting BI, or start BI and change the catheter, flush the catheter and if that does not help, rule out the presence of a stone or bladder over activity or only advising to increase fluid intake. Most RDs (*n* = 6, 42.9%) stated to take a UC and wait. In all, 26 urologists (74.3%) stated not changing the catheter. RDs were divided, half of them stated not to change the catheter and half of them would.

One RD stated to leave treatment to the GP or urologist in both scenarios of the first case and to ask the GP to perform a culture and start antibiotics in all three scenarios of the second case.

#### Nurses

In the first scenario of the first case, most nurses from the urology department (*n* = 31, 75.6%) and rehabilitation centre/department (*n* = 8, 66.7%) stated to take a UC and consult the physician for further actions. Two nurses from the urology department stated to also evaluate the fluid intake of the patient and one would check CIC frequency and technique.

In the second scenario, again, most nurses from urology departments (*n* = 23, 57.5%) and rehabilitation centres/departments (*n* = 7, 58.3%) stated to take a UC and consult the physician for further actions. Seven nurses (17.5%) from urology departments gave other answer options, all stated to evaluate the fluid intake, CIC technique, the residue and consult the physician if necessary. One nurse from a rehabilitation centre/department stated to directly start with antibiotics and change if necessary when the results of the culture are available.

In the first scenario of the second case, again most nurses from urology departments (*n* = 29, 76.3%) and rehabilitation centres/departments (*n* = 8, 80%) stated to take a UC and consult the physician for further actions. Two nurses from the urology department and one from the rehabilitation centre/department answered changing the catheter as a treatment option and one would flush the catheter. Most nurses would change the catheter in this situation.

In the second situation, answers were more divided. In all, 10 (27%) nurses from urology departments stated to take a culture and wait, 10 (27%) to take a culture and consult the physician and seven (18.9%) gave a different answer option: two would advise increased fluid intake, one would flush the catheter, one would advise to flush the catheter and start BI, two would give a prescription for antibiotics after consulting the physician and one would start antibiotics and change the catheter during treatment. Half of the nurses (*n* = 5) from rehabilitation centres/departments stated to take a UC and consult a physician. Most nurses stated not changing the catheter in this situation.

In the last scenario, again answers were divided. Almost a quarter (*n* = 9, 23.7%) of nurses from urology departments stated to take a culture and wait, and almost a quarter (*n* = 9, 23.7%) stated to schedule a consultation with a nurse at the outpatient clinic. Six nurses gave a different answer option, five would check the catheter for blockage and change the catheter if necessary and one would start antibiotics and change the catheter during treatment. A fifth (*n* = 2) of the nurses from rehabilitation centres/departments stated to wait, a fifth to take a culture and consult the physician and a fifth would refer the patient to their GP/treating physician. Three nurses gave different answer options, two would start BI and one would change the catheter and advise increased fluid intake. Slightly more than half of the nurses from urology departments and 70% of the nurses from rehabilitation centres/departments stated to not change the catheter in this scenario.

#### Medical assistants

All medical assistants of urology departments and GP offices stated to consult a physician for further actions, or schedule an appointment with a physician and do a urine analysis in all scenarios. The open-answer option was also often used. In the first situation of case one, three medical assistants of urology departments stated to do a urine analysis and, if necessary, consult the physician for further actions afterwards and two would evaluate if the patients had enough fluid intake or advise to increase their fluid intake. In the second scenario two medical assistants of urology departments would measure the bladder residue, do a urine analysis and consult the physician afterwards for further actions, one would schedule an appointment with the nurse for further evaluation and one would advise the patient to increase his/her fluid intake and consult the physician if antibiotics should be started.

In the first scenario of the second case, one medical secretary/assistant of a urology department stated to take a culture and wait for results and one to take a culture and consult the physician if antibiotics should be started. In the second scenario, two medical assistants of urology departments stated to do a culture instead of urine analysis and consult the physician afterwards and one stated to do a urine analysis at the outpatient clinic. In the third scenario, two medical assistants of urology departments stated to check the catheter for blockage and consult the physician for medication for bladder cramping and one stated to do a urine analysis and only consult the physician if necessary.

One medical assistant from the GP office stated to do a urine analysis and consult the urologist if one is involved, or plan a visit with the GP if necessary, in all situations of both cases.

### Guidelines

Participants were asked if there was a clear definition of CAUTIs, that their department complied with. In total, 16 (45.7%) urologists, 10 (71.4%) RDs and one (50%) GP stated that such a definition was available at their department, 10 urologists (28.6%) and one (7.1%) RD stated possibly, but I am not aware of it and nine urologists (25.7%), three (21.4%) RDs and one (50%) GP stated that such definition was not available.

Of continence nurses working at urology departments, 12 (31.6%) said a clear definition of CAUTI was available at their department, 20 (52.6%) answered possible, but I am not aware of it and six (15.8%) stated no definition was available. Three (30%) continence nurses working at a rehabilitation centre/department indicated a definition was available, five (50%) answered possible, but I am not aware of it and two (20%) stated no definition was available.

Five (62.5%) medical assistants working in urology departments answered that there was a clear definition of CAUTI available, two (25%) answered possible, but I am not aware of it and one (12.5%) said no definition was available. Two (66.6%) medical assistants working at GP offices stated a clear definition was available and one (33.3%) said no definition was available.

An overview of the used guidelines is shown in Table 5. Most urologists (*n* = 15, 34.1%) used the European Association of Urology (EAU) guideline. More than a third (*n* = 6, 37.5%) of the RDs used the definition from the multidisciplinary guideline ‘Neurogenic bladder’. One GP (100%) and two (66.6%) medical secretaries/assistants working at GP offices stated to use the definition from the Dutch GP Association guideline (Nederlands Huisartsen Genootschap, NHG).

**Table 5.** Guidelines used for the definition of CAUTIs per healthcare workers involved.

Name of association/guideline	Urologists (*n* = 44)	Continence nurses urology department (*n* = 44)	Medical secretaries/assistants urology department (*n* = 5)	RDs (*n* = 16)	Continence nurse’s rehabilitation medicine (*n* = 12)
European Association of Urology (EAU)	*n* = 15 (34.1%)	*n* = 6 (13.6%)	*n* = 0	*n* = 2 (12.5%)	*n* = 1 (8.3%)
Dutch Urological Association (Nederlandse Vereniging van Urologie, NVU)	*n* = 11 (25%)	*n* = 6 (13.6%)	*n* = 0	*n* = 1 (6.3%)	*n* = 1 (8.3%)
Dutch Association of Rehabilitation Doctors (Vereniging van Revalidatieartsen, VvRA)	*n* = 0	*n* = 0	*n* = 0	*n* = 2 (12.5%)	*n* = 0
International Spinal Cord Society (ISCoS)	*n* = 1 (2.3%)	*n* = 0	*n* = 0	*n* = 1 (6.3%)	*n* = 1 (8.3%)
Multidisciplinary guideline ‘Neurogenic bladder’, written by NVU, NVN, VvRA and Verenso	*n* = 4 (9.1%)	*n* = 2 (4.5%)	*n* = 0	*n* = 6 (37.5%)	*n* = 1 (8.3%)
Antibiotic guideline workgroup foundation (Stichting Werkgroep Antibiotica Beleid, SWAB)	*n* = 3 (6.8%)	*n* = 1 (2.3%)	*n* = 0	*n* = 2 (12.5%)	*n* = 0
Dutch GP Association (Nederlands Huisartsen Genootschap, NHG)	*n* = 1 (2.3%)	*n* = 1 (2.3%)	*n* = 0	*n* = 1 (6.3%)	*n* = 0
European Association of Urology Nurses (EAUN)	*n* = 0	*n* = 3 (6.8%)	*n* = 0	*n* = 0	*n* = 0
The Dutch Association of Nurses (CV&V)	*n* = 0	*n* = 1 (2.3%)	*n* = 0	*n* = 0	*n* = 0
The Dutch Flemish Spinal Cord Injury Association (Nederlands Vlaams Dwarslaesie Genootschap, NVDG)	*n* = 0	*n* = 0	*n* = 0	*n* = 0	*n* = 1 (8.3%)
National Expertise Network Continence Nurses SCI Centres (Landelijk Expertisenetwerk Continentieverpleegkundigen Dwarslaesiecentra, LECD)	*n* = 0	*n* = 0	*n* = 0	*n* = 0	*n* = 1 (8.3%)
Not sure which guidelines is used for the definition	*n* = 9 (20.5%)	*n* = 24 (54.5%)	*n* = 5 (100%)	*n* = 1 (6.3%)	*n* = 6 (50%)

Most nurses from urology departments (54.4%) and rehabilitation centres (50%) and all (*n* = 5) medical assistants from urology departments stated not to be sure from which guideline their used definition originated.

A urologist and a continence nurse working at urology departments stated that a definition will never be clear, because patients on CIC or with an IC suffer from asymptomatic bacteriuria and will never have sterile/clean urine.

## Discussion

In this survey, we obtained an insight into the variation in clinical practice in the definition of CAUTIs and treatment policies, between healthcare workers involved. As we hypothesized, a considerable variation between the used definition of CAUTIs and following therapeutic choices was seen between the participants of the survey. These differences were seen between different departments but also within subspecialties. The fact that the open answer option ‘other, namely’ was used often in all questions and clinical scenarios can be explained by the disunity between the healthcare workers involved. The results are in line with previous reported literature. Forster *et al.*^9^ previously published results of a survey among healthcare workers regarding the diagnosis of UTI in children with neurogenic bladders who require CIC and reported variability too. They concluded that standardization and implementation of consensus criteria for UTI in this high-risk population is needed. A systematic review performed by Madden-Fuentes *et al*.^11^ showed that used definitions of UTI in spina bifida patients are heterogeneous and advocate a standard definition for this population. A survey performed almost 20 years ago on bacteriuria management in patients with spina bifida, showed no consensus at clinics that specialize in the care of these patients and indicated a clear need for evidence-based guidelines to assist healthcare workers in their medical decision-making.^12^ As Forster *et al.*^9^ noticed, most research is aimed at urologists, but diagnosing and treating CAUTIs involves far more caretakers, which our survey confirms. It starts when a patients reaches out to the treating department and triage is performed by assisting healthcare workers/nurses and their assessment if consulting a physician is necessary.

Unfortunately, participants were not equally distributed between the different included subspecialties (urology, rehabilitation medicine and GP). The majority of participants were working in the field of urology. A possible explanation could be that the difficulty of diagnosing a CAUTI is more common within the urologic patient population and healthcare workers from urology departments feel a stronger urge to contribute in research regarding this issue. A previously conducted survey among GPs in the Netherlands on guideline adherence of asymptomatic bacteriuria had a greater response rate. Results showed that most GPs followed their national guideline regarding UTIs, but knowledge about asymptomatic bacteriuria could be improved.^13^ Not all RDs are involved in a patient population that requires an IC or CIC which can explain a lower number of respondents in this field.

Almost all participants stated to see patients with an IC or on CIC on a quite regular basis and their experience can be qualified as sufficient. It is known from a previous study among nurses, that knowledge, but more so attitude plays an essential role in CAUTI prevention, and education and training are advised.^14^

In the clinical scenarios of case two, a woman with a suprapubic IC, participants were asked about if they would change the catheter. Again, answers differed quite strongly between urologists, RDs and continence nurses from the urology/rehabilitation departments. Changing catheters, when a CAUTI is suspected or is proven, is a well-known point of debate in current literature and clinical benefit is still not confirmed.^15^ The EAU guideline and guideline of infectious diseases of America state that a catheter replacement is recommended, based on one reference of Razz *et al.*^16^ The authors performed a prospective randomized controlled trial in patients with long-term ICs and recommend catheter replacement in patients with a symptomatic CAUTI, but an adequate power analysis is lacking. A multidisciplinary guideline with a clear and clinical-minded approach is advised, but more research on this topic is needed. The same applies to BI for prevention or as a treatment for (symptoms of) CAUTIs,^17^ and our research showed inconsistent usage of BI, reasons for starting BI and used substance.

When participants were asked about guidelines they used for the definition of CAUTI, again, a large variety of answers were given, and a total of 10 different guidelines were named. Also, a great number of participants stated not to be aware if a certain guideline was used or available at their department or no guideline was available. The lack of uniformity has been shown in earlier studies.^18^ Several guidelines are available, as can be seen in our results, but not all are widely known and incorporated in clinical practice.

This study has some limitations, not all departments are represented evenly and the lack of participating GPs leaves a shortage of information. The questions of this survey were developed by the research group, which is urology-oriented. Although an independent urologist was consulted, the questionnaires were not validated, which might have led to bias. Overall this survey had a sufficient number of participants and confirmed the hypothesis that there is a lack of uniformity and knowledge regarding CAUTIs.

More research regarding the development of a comprehensive definition and guideline for CAUTIs that is suitable for all subspecialties and healthcare workers, involving all departments that are responsible for the care of patients with an IC or on CIC, is necessary.

## Conclusion

This survey showed considerable variation in the clinical practice of diagnosing and managing CAUTIs between the healthcare workers involved. Uniformity in the management of CAUTIs, to prevent treatment of asymptomatic bacteriuria and possible resistance to antibiotics in these high-risk patients, is advised. Preferably, this guideline transcends departments so that every patient with a CAUTI is treated similarly and overtreatment is prevented.

## Supplemental Material

sj-docx-1-tau-10.1177_17562872231191305 – Supplemental material for Variation of diagnosis and treatment of catheter-associated urinary tract infections: an online survey among caretakers involved Click here for additional data file. Supplemental material, sj-docx-1-tau-10.1177_17562872231191305 for Variation of diagnosis and treatment of catheter-associated urinary tract infections: an online survey among caretakers involved by Tess van Doorn, Sophie A. Berendsen, Rosa L. Coolen, Jeroen R. Scheepe and Bertil F. M. Blok in Therapeutic Advances in Urology

sj-docx-2-tau-10.1177_17562872231191305 – Supplemental material for Variation of diagnosis and treatment of catheter-associated urinary tract infections: an online survey among caretakers involved Click here for additional data file. Supplemental material, sj-docx-2-tau-10.1177_17562872231191305 for Variation of diagnosis and treatment of catheter-associated urinary tract infections: an online survey among caretakers involved by Tess van Doorn, Sophie A. Berendsen, Rosa L. Coolen, Jeroen R. Scheepe and Bertil F. M. Blok in Therapeutic Advances in Urology

## Declarations

**Ethics approval and consent to participate:** The Daily Board of the Medical Ethics Committee Erasmus MC of Rotterdam, The Netherlands, has reviewed the research proposal of this survey (study ID MEC-2021-0156). As a result of this review, the Committee concluded that the rules laid down in the Medical Research Involving Human Subjects Act, do not apply to this research proposal. Participants were aware of the anonymous nature of this survey and gave their consent to use the collected data by participating.

**Consent for publication:** Not applicable.

**Availability of data and materials:** The dataset used and analysed during the current study is available from the corresponding author upon reasonable request.
