# Supplementary material for: Variation of diagnosis and treatment of catheter-associated urinary tract infections: an online survey among caretakers involved
Source: Ther Adv Urol. 2023 Sep 6;15:17562872231191305. doi: 10.1177/17562872231191305 (PMC10483975; doi:10.1177/17562872231191305)
Supplement: sj-docx-1-tau-10.1177_17562872231191305 – Supplemental material for Variation of diagnosis and treatment of catheter-associated urinary tract infections: an online survey among caretakers involved [file sj-docx-1-tau-10.1177_17562872231191305.docx]

**Supplementary file 1: questionnaires used for the survey.**

**Doctors:**

1. What is your working field?

a) General practitioner

b) Rehabilitation medicine

c) Urology

3. Do you work in a general or university hospital? (question is not asked when answer option GP is given in the previous question)

a) General hospital

b) University hospital

c) Other, namely …..

4. How long have you been working in your field?

a) < 5 years

b) 5 – 10 years

c) 10 – 15 years

d) > 15 years

5. How often do you see patients at your practice/outpatient clinic who are on clean intermittent catheterization?

a) Daily

b) Weekly

c) Monthly

d) Annually

e) Never

6. How often do you see patients at your practice/outpatient clinic who have an indwelling catheter?

a) Daily

b) Weekly

c) Monthly

d) Annually

e) Never

7. Do you ever start bladder irrigation in patients with (possible) urinary tract infections?

a) Yes

b) No

8. what is the main reason to start bladder irrigation ?

a) Prevention of CAUTI’s

b) Treatment of CAUTI’s

c) Treatment of symptoms

d) Treatment of blockage of the catheter

e) Other, namely …..

9. What substance do you advise to irrigate the bladder with? (multiple answers are correct)

a) Tap water

b) NaCl

c) Solutio G/Solutio R

d) GAG-layer repearing irrigation (Gepan, Elmiron)

e) Gentamycine

f) Povidon iode

g) Something else, namely …..

10. Is there a clear definition of a urinary tract infection in patients with intermittent catheterization or with an indwelling catheter in your department/within your department?

a) Yes

b) No

c) Possibly, but I am not aware of it

11. If this definition is based on a professional guideline, the guideline of which association is used?

a) European Association of Urology (EAU)

b) Dutch Urological Association (Nederlandse Vereniging van Urologie, NVU)

c) Dutch Assocication of rehabilitation doctors (Vereniging van Revalidatieartsen, VvRA)

d) International Spinal Cord Society (ISCoS)

e) Multidisciplinary guideline ‘Neurogenic bladder’, written by NVU, NVN, VvRA and Verenso

f) Antibiotic guideline workgroup foundation (Stichting Werkgroep Antibiotica Beleid, SWAB

g) Dutch GP Association (Nederlands Huisartsen Genootschap, NHG)

h) European Association of Urology Nurses (EAUN)

I) Other, namely …..

**Nurses:**

1. What is your working field?

a) Rehabilitation medicine

b) Urology

2. Do you work in a rehabilitation center, general or university hospital?

a) Rehabilitation Center

b) General hospital

c) University Hospital

e) Other, namely ……

4. How long have you been working in your field?

a) < 5 years

b) 5 – 10 years

c) 10 – 15 years

d) > 15 years

5. How often do you see patients at your practice/outpatient clinic who are on clean intermittent catheterization?

a) Daily

b) Weekly

c) Monthly

d) Annually

e) Never

6. How often do you see patients at your practice/outpatient clinic who have an indwelling catheter?

a) Daily

b) Weekly

c) Monthly

d) Annually

e) Never

7. Do you (or the physician) ever start bladder irrigation in patients with (possible) urinary tract infections?

a) Yes

b) No

8. what is the main reason to start bladder irrigation?

a) Prevention of CAUTI’s

b) Treatment of CAUTI’s

c) Treatment of symptoms

d) Treatment of blockage of the catheter

e) Other, namely …..

9. What do you (or the physician) advise to irrigate the bladder with? (multiple answers are correct)

a) Tap water

b) NaCl

c) Solutio G/Solutio R

d) GAG-layer repearing irrigation (Gepan, Elmiron)

e) Gentamycine

f) Povidon iode

g) Something else, namely …….

10. Is there a clear definition of a urinary tract infection in patients with intermittent catheterization or with an indwelling catheter in your department/within your department?

a) Yes

b) No

c) Possibly, but I am not aware of it

11. If this definition is based on a professional guideline, the guideline of which association is used?

a) European Association of Urology (EAU)

b) Dutch Urological Association (Nederlandse Vereniging van Urologie, NVU)

c) Dutch Assocication of rehabilitation doctors (Vereniging van Revalidatieartsen, VvRA)

d) International Spinal Cord Society (ISCoS)

e) Multidisciplinary guideline ‘Neurogenic bladder’, written by NVU, NVN, VvRA and Verenso

f) Antibiotic guideline workgroup foundation (Stichting Werkgroep Antibiotica Beleid, SWAB

g) Dutch GP Association (Nederlands Huisartsen Genootschap, NHG)

h) European Association of Urology Nurses (EAUN)

I) Other, namely…..

**Medical assistants**

1. What is your working field?

a) Rehabilitation medicine

b) Urology

2. Do you work in a rehabilitation center, general or university hospital?

a) Rehabilitation Center

b) General hospital

c) University Hospital

e) Other, namely ……

4. How long have you been working in your field?

a) < 5 years

b) 5 – 10 years

c) 10 – 15 years

d) > 15 years

5. How often do you see patients at your practice/outpatient clinic who are on clean intermittent catheterization?

a) Daily

b) Weekly

c) Monthly

d) Annually

e) Never

6. How often do you see patients at your practice/outpatient clinic who have an indwelling catheter?

a) Daily

b) Weekly

c) Monthly

d) Annually

e) Never

7. Is there a clear definition of a urinary tract infection in patients with intermittent catheterization or with an indwelling catheter in your department/within your department?

a) Yes

b) No

c) Possibly, but I am not aware of it

8. If this definition is based on a professional guideline, the guideline of which association is used?

a) European Association of Urology (EAU)

b) Dutch Urological Association (Nederlandse Vereniging van Urologie, NVU)

c) Dutch Assocication of rehabilitation doctors (Vereniging van Revalidatieartsen, VvRA)

d) International Spinal Cord Society (ISCoS)

e) Multidisciplinary guideline ‘Neurogenic bladder’, written by NVU, NVN, VvRA and Verenso

f) Antibiotic guideline workgroup foundation (Stichting Werkgroep Antibiotica Beleid, SWAB

g) Dutch GP Association (Nederlands Huisartsen Genootschap, NHG)

h) European Association of Urology Nurses (EAUN)

I) Other, namely…..
