# Supplementary material for: Variation of diagnosis and treatment of catheter-associated urinary tract infections: an online survey among caretakers involved
Source: Ther Adv Urol. 2023 Sep 6;15:17562872231191305. doi: 10.1177/17562872231191305 (PMC10483975; doi:10.1177/17562872231191305)
Supplement: sj-docx-2-tau-10.1177_17562872231191305 – Supplemental material for Variation of diagnosis and treatment of catheter-associated urinary tract infections: an online survey among caretakers involved [file sj-docx-2-tau-10.1177_17562872231191305.docx]

Supplementary file 2: table with all collected outcome measures.

| Characteristics of participants (table 2) | Working environment |
| --- | --- |
|  | Working experience |
|  | Incidence of seeing patients with on CIC |
|  | Incidence of seeing patients with an IC |
| Bladder irrigation (table 3) | Starting bladder irrigation |
|  | Reasons to start bladder irrigation |
|  | Substance used to irrigate the bladder with |
| Clinical scenario’s (table 4) | Next step in clinical practice |
|  | Changing catheter or not |
| Guidelines used (table 5) | All mentioned guidelines |
